# Adoption and Implementation of Evidence-Based Colorectal Cancer Screening Interventions Among Cancer Control Program Grantees, 2009–2015

**DOI:** 10.5888/pcd16.180682

**Published:** 2019-10-10

**Authors:** Peggy A. Hannon, Annette E. Maxwell, Cam Escoffery, Thuy Vu, Marlana J. Kohn, Lindsay Gressard, Laurel Dillon-Sumner, Caitlin Mason, Amy DeGroff

**Affiliations:** 1 University of Washington, Seattle, Washington; 2 University of California, Los Angeles, California; 3 Emory University, Atlanta, Georgia; 4 Centers for Disease Control and Prevention, Atlanta, Georgia

## Abstract

**Purpose and Objectives:**

Colorectal cancer (CRC) is the second-leading cause of cancer death in the United States. Although effective CRC screening tests exist, CRC screening is underused. Use of evidence-based interventions (EBIs) to increase CRC screening could save many lives. The Colorectal Cancer Control Program (CRCCP) of the Centers for Disease Control and Prevention (CDC) provides a unique opportunity to study EBI adoption, implementation, and maintenance. We assessed 1) the number of grantees implementing 5 EBIs during 2011 through 2015, 2) grantees’ perceived ease of implementing each EBI, and 3) grantees’ reasons for stopping EBI implementation.

**Intervention Approach:**

CDC funded 25 states and 4 tribal entities to participate in the CRCCP. Grantees used CRCCP funds to 1) provide CRC screening to individuals who were uninsured and low-income, and 2) promote CRC screening at the population level. One component of the CRC screening promotion effort was implementing 1 or more of 5 EBIs to increase CRC screening rates.

**Evaluation Methods:**

We surveyed CRCCP grantees about EBI implementation with an online survey in 2011, 2012, 2013, and 2015. We conducted descriptive analyses of closed-ended items and coded open-text responses for themes related to barriers and facilitators to EBI implementation.

**Results:**

Most grantees implemented small media (≥25) or client reminders (≥21) or both all program years. Although few grantees reported implementation of EBIs such as reducing structural barriers (n = 14) and provider reminders (n = 9) in 2011, implementation of these EBIs increased over time. Implementation of provider assessment and feedback increased over time, but was reported by the fewest grantees (n = 17) in 2015. Reasons for discontinuing EBIs included funding ending, competing priorities, or limited staff capacity.

**Implications for Public Health:**

CRCCP grantees implemented EBIs across all years studied, yet implementation varied by EBI and did not get easier with time. Our findings can inform long-term planning for EBIs with state and tribal public health institutions and their partners.

**Summary**

**What is already known on this topic?**

Colorectal Cancer Control Program (CRCCP) grantees from 2009 through 2015 were encouraged to implement evidence-based interventions (EBIs) to promote colorectal cancer screening.

**What is added by this report?**

This report studies EBI implementation over a 5-year period in a stable group of grantees.

**What are the implications for public health practice?**

There was some turnover regarding which EBIs were implemented, and implementation did not get easier over time for EBIs that were sustained. Our findings can be applied to evaluating and supporting EBI implementation in the next CRCCP funding cycle and in the National Breast and Cervical Cancer Early Detection Program as they adopt a similar approach to promoting EBIs and collaborating with health systems.

## Introduction

Colorectal cancer (CRC) is the second-leading cause of cancer death in the United States (1). The US Preventive Services Task Force (USPSTF) recommends CRC screening for average risk adults, aged 50 to 75 years, using either stool-based tests (ie, fecal occult blood test [FOBT], fecal immunochemical test [FIT], or multitargeted stool DNA test [FIT-DNA]) or tests that directly visualize the colon (ie, colonoscopy, sigmoidoscopy, or computed tomographic colonography) (2). However, CRC screening is underused; estimates of screening rates in the United States range from 63% (National Health Interview Survey, 2015) to 68% (Behavioral Risk Factor Surveillance System, 2016) (3,4). CRC screening rates are substantially lower for populations without health insurance, populations without a medical home, and Asian and Pacific Islander and Hispanic populations (3,5,6). Increasing CRC screening rates to 80% has the potential to prevent 277,000 CRC cases and 203,000 CRC deaths by 2030 (3), partly because CRC screening has the potential not only to detect cancer early but also to prevent it through the identification and removal of precancerous polyps. Many organizations support the “80% in Every Community” initiative (http://nccrt.org/80-in-every-community/), which established the goal of 80% of the total population of adults aged 50 to 75 years being up to date for CRC screening.

The Colorectal Cancer Control Program (CRCCP) is a CDC initiative to increase CRC screening among adults aged 50 to 75 (www.cdc.gov/cancer/crccp) (7). The program’s grantees — most often state health departments and tribal health agencies — are funded (in part) to promote CRC screening by using 5 evidence-based interventions (EBIs). We focus on the first cycle of the CRCCP from 2009 through 2015, which funded 29 grantees. The evaluation activities we describe were conducted from 2011 through 2015.

## Purpose and Objectives

The CRCCP provides a unique opportunity to study EBI adoption, implementation, and maintenance over several years in a stable group of grantee organizations and in the context of a national program. Few studies examine EBI implementation among the same organizations longitudinally over the course of 5 years or more (8,9). We studied grantees’ early experiences with adopting and implementing EBIs and compared their experiences with National Breast and Cervical Cancer Early Detection Program (NBCCEDP) grantees that did not receive CRCCP funding and were not explicitly directed to use EBIs (10–13). Grantees and nongrantees were equally likely to implement practices that are not recommended by the Guide to Community Preventive Services (Community Guide), but grantees were more likely to implement EBIs (13). This finding showed that CDC’s encouragement and financial support to the grantees to use these EBIs was effective, because all grantees were using at least 1 or 2 EBIs by the end of the second program year. The intended contribution of this study is to determine whether grantees maintained the EBIs they implemented over time and why (or why not). We assessed 1) how many grantees implemented each EBI from 2009 through 2015; 2) grantees’ perceived ease of implementing each EBI; 3) the maintenance of specific EBIs from year to year; and 4) qualitative data describing why grantees stopped using EBIs as well as facilitators and barriers to implementing EBIs.

## Intervention Approach

CDC’s Division of Cancer Prevention and Control initially funded the CRCCP in 2009. The overall goal of CRCCP was to increase CRC screening rates to 80% in funded states and tribal areas by the end of the funding cycle, with a long-term objective of reducing CRC incidence and mortality. In 2009, a total of 22 states and 4 tribal entities were awarded CRCCP funds; an additional 3 states received CRCCP funds in 2010. All grantees’ awards lasted through June 2015 (7).

Grantees used CRCCP funds for 2 program components. First, grantees provided CRC screening services to low-income and uninsured people in their region. Second, grantees promoted CRC screening at the population level. Grantees were strongly encouraged to use 1 or more of 5 EBIs from the Community Guide to promote CRC screening (grantees were free to choose any combination of the EBIs to implement and could change their choices over time). The Community Guide conducts systematic reviews of evidence to identify effective strategies to increase cancer screening and other health desirable behaviors (14). Three of the EBIs are classified as “client-oriented,” meaning they focus on the person needing screening; these EBIs are small media (such as brochures, postcards, or posters), client reminders, and reducing structural barriers. Two of the EBIs are classified as “provider-oriented,” meaning they increase the likelihood that providers will recommend screening; these EBIs are provider reminders and provider assessment and feedback (15,16). In addition, CDC encouraged grantees to use patient navigation; the NIH state-of-the-science conference statement on enhancing the use and quality of CRC screening recommends patient navigation as an evidence-based strategy for CRC screening (17).

The EBIs listed above vary in terms of complexity and partnerships required. The client-oriented EBIs could be implemented directly by grantees or by their clinical or community partners. The provider-oriented EBIs may be more complex from the perspective of a typical grantee organization because they require 1 or more clinic or health system partners. In addition, implementing provider reminders or provider assessment and feedback may require working with or adapting electronic health records. Given the grantees’ organizational context (state and tribal departments of health), the provider-oriented EBIs may be more challenging to implement than the client-oriented EBIs.

A key assumption underlying the CRCCP is that if grantees implement EBIs, CRC screening rates will increase. The evaluation described below focused on whether grantees implemented and maintained EBIs over the funding cycle (measured with quantitative survey items) and barriers and facilitators to implementing and maintaining EBIs (measured with open-text survey responses).

## Evaluation Methods

Staff members of CDC and the Cancer Prevention and Control Research Network (CPCRN) conducted this study. CPCRN is a national network of academic, public health, and community partners who work together to reduce the burden of cancer, especially among those disproportionately affected (18–20). CDC and the National Cancer Institute (NCI) fund the CPCRN to accelerate the adoption of evidence-based cancer prevention and control practices.

A CPCRN work group collaborated with CDC to develop and implement a grantee survey as part of the CRCCP evaluation. The first online survey of the 29 CRCCP grantees asked about the first 2 years of program implementation and was administered during November and December 2011. Subsequent grantee surveys were administered in 2012 (program year 3), 2013 (program year 4), and 2015 (program year 6). The survey was administered following the end of each fiscal year. No survey was administered for program year 5 because of delays with the Paperwork Reduction Act review process.

Grantee organizations were 25 state departments of health and 4 tribal organizations that received funding through the CRCCP. For every survey administration, the 29 CRCCP program directors received an emailed invitation letter jointly signed by CDC and the CPCRN asking them to identify the person most knowledgeable about day-to-day operations of the CRCCP to complete the survey. Typically, this was a program director or coordinator. Respondents completed the survey online; the process was programmed by using Qualtrics survey software (Qualtrics) in 2011 and by using DatStat Illume survey software (DatStat Corp) in 2012 through 2015. The survey questionnaire and procedures were declared exempt from review by the University of Washington and CDC institutional review boards. Data collection was approved by the Office of Management and Budget (control number 0920–1074).

### Survey questionnaire

The questionnaire covered several topics; we present data on grantee efforts to promote population-level CRC screening for the first funding cycle of CRCCP (2009–2015). The survey included questions about grantee organization type (state department of health or tribal organization), survey respondent characteristics (role in CRCCP, length of involvement in CRCCP, and length of involvement in cancer control), whether there was turnover in the program director or program manager roles during 2009 through 2015, and questions about use of each of the 5 Community Guide–recommended EBIs. For each EBI, respondents were asked whether their CRCCP currently uses it or plans to use it in the next 12 months. In the 2012 through 2015 administrations of the survey, grantees were also asked if they implemented each EBI in the past but no longer do so.

Respondents rated the ease of implementing the EBIs on a 5-point Likert scale (1 = very difficult, 5 = very easy). They then answered open-ended questions specifically about facilitators and barriers to implementing EBIs. Grantees could also add any comments about EBI implementation that they did not provide earlier (eg, facilitators and barriers, success stories, and, if applicable, reasons for no longer using an EBI). For each survey administration, the questionnaire was pilot-tested with 4 grantees; 2 grantees reviewed a paper version of the questionnaire, and 2 reviewed the online version. The final questionnaire was revised to address feedback from the pilot test. Survey items are available from the authors on request.

### Data analysis

All quantitative data were analyzed by using SPSS version 18 (IBM Inc). We performed descriptive analyses to determine the frequency of CRCCP grantees’ use of EBIs, mean ratings of “ease of implementing” EBIs, and frequency of grantees’ discontinuing EBIs. Two coders (L.D.S., P.A.H.) did a content analysis of grantees’ open-text responses about facilitators and barriers to use of EBIs, and reasons for discontinuing EBIs. One coder (L.D.S.) did initial development of the codebook by using an emergent coding approach. The other coder (P.A.H.) reviewed the codebook and initial codes; the coders discussed and resolved discrepancies. Grantees had the opportunity to provide open-text responses about each EBI in each program year. Responses were first coded separately by EBI and program year. The same themes came up across EBIs and program years, so the coders aggregated the results.

## Results

### Survey respondents

Almost all grantees participated in all 4 of the surveys; 28 grantees (96%) completed the survey in 2011 and 2013, and 29 grantees (100%) completed the survey in 2012 and 2015. Most survey respondents (82%) in 2015 were the program director and/or the program manager (Table 1).

**Table 1:** Grantee and Survey Respondent Characteristics (N = 29), Colorectal Cancer Control Program (CRCCP), 2015

Characteristic	No. (%)
**Grantee organization type**	
State department of health	25 (86)
Tribal organization	4 (14)
**Respondent role in CRCCP**	
Program director	12 (41)
Program manager	9 (31)
Program director and manager	3 (10)
Other	5 (17)
**Length of respondent’s involvement in CRCCP**	
<1 year	4 (14)
12–23 months	3 (10)
24–35 months	4 (14)
≥3 years	18 (62)
**Length of respondents’ involvement in cancer control, y**	
<1	2 (7)
1–3	4 (14)
4–5	2 (7)
≥6	21 (72)
**Change in CRCCP’s program director or program manager during 2009–2015**	
Yes, the program manager changed	5 (17)
Yes, the program director changed	6 (21)
Yes, both changed	6 (21)
No, there has been no change in either the program director or program manager	12 (41)

### EBI adoption, implementation, and maintenance

Overall, more grantees implemented client-oriented EBIs than provider-oriented EBIs (Table 2). By 2015 (program year 6), most grantees were implementing small media (n = 25) and client reminders (n = 26); few grantees stopped implementing these EBIs by 2015 (n = 4 for small media and n = 2 for client reminders) (Figure). Reducing structural barriers is classified as a client-oriented EBI, yet often requires health system support. In 2011, 14 grantees implemented reducing structural barriers, increasing to 23 by 2015. Fewer grantees implemented the provider-oriented EBIs, especially in the first years of the funding cycle. In 2011, 9 grantees implemented provider reminders and 14 implemented provider assessment and feedback. By 2015, 19 grantees implemented provider reminders and 17 implemented provider assessment and feedback. We saw the most turbulence for provider assessment and feedback; 9 grantees that reported implementing this EBI in program year 2 reported they no longer did so in program year 3.

**Table 2:** Evidence-based Intervention (EBI) Implementation, Ease of EBI Implementation,^a^ and EBI Maintenance,^b^ Colorectal Cancer Control Program, 2011–2015

Category	Program Year 2, 2011 (n = 28)	Program Year 3, 2012 (n = 29)	Program Year 4, 2013 (n = 28)	Program Year 6, 2015 (n = 29)
**Small media**				
**No. of grantees implementing**	**27**	**28**	**28**	**25**
No. grantees maintaining implementation	—	26	27	24
No. grantees discontinuing implementation	—	1	0	4
No. grantees newly implementing	—	1	1	0
**Average ease of implementation (SD)**	**4.15 (1.08)**	**3.65 (0.75)**	**3.92 (0.80)**	**3.92 (0.86)**
**Client reminders**				
**No. of grantees implementing**	**21**	**22**	**23**	**26**
No. grantees maintaining implementation	—	19	19	21
No. grantees discontinuing implementation	—	2	2	2
No. grantees newly implementing	—	3	4	4
**Average ease of implementation (SD)**	**3.95 (0.74)**	**3.50 (1.03)**	**3.29 (0.92)**	**3.31 (1.12)**
**Reducing structural barriers**				
**No. of grantees implementing**	**14**	**17**	**23**	**23**
No. grantees maintaining implementation	—	10	15	20
No. grantees discontinuing implementation	—	4	1	3
No. grantees newly implementing	—	6	8	2
**Average ease of implementation (SD)**	**3.43 (1.16)**	**3.20 (1.08)**	**3.18 (0.96)**	**3.09 (1.00)**
**Provider reminders**				
**No. of grantees implementing**	**9**	**11**	**19**	**19**
No. grantees maintaining implementation	—	6	8	16
No. grantees discontinuing implementation	—	3	3	3
No. grantees newly implementing	—	5	11	2
**Average ease of implementation (SD)**	**3.56 (0.73)**	**3.40 (1.26)**	**2.47 (0.83)**	**3.26 (1.10)**
**Provider assessment and feedback**				
**No. grantees implementing**	**14**	**13**	**15**	**17**
No. grantees maintaining implementation	—	5	9	13
No. grantees discontinuing implementation	—	9	3	2
No. grantees newly implementing	—	7	6	4
**Average ease of implementation rating (SD)**	**3.71 (1.14)**	**3.10 (1.20)**	**1.92 (0.52)**	**2.53 (1.33)**

Abbreviation: — , not applicable; SD, standard deviation.

a Respondents rated the ease of implementing the EBIs on a 5-point Likert scale (1 = very difficult, 5 = very easy).

b Maintenance is defined as responding, “Yes, we currently implement this EBI” in 2 consecutive administrations of this survey. In a few cases, grantees maintaining implementation could not be computed for a given grantee because they did not complete the grantee survey for the prior year. In these cases, the numbers for grantees maintaining implementation, grantees discontinuing implementation, and grantees newly implementing will sum to less than the total grantees implementing number for a given program year.

**Figure:**
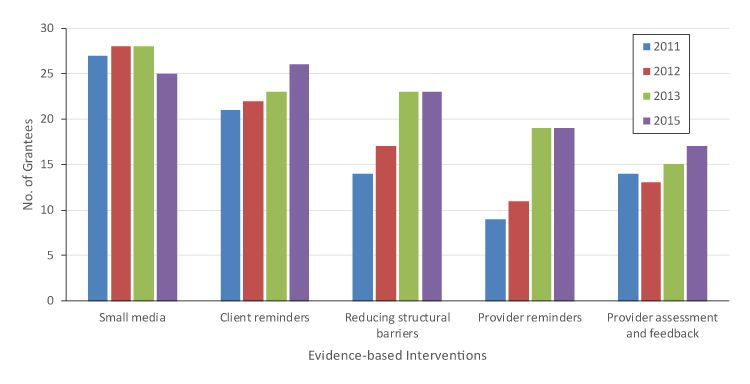
Number of grantees implementing evidence-based interventions among grantees for the Colorectal Cancer Control Program, 2011–2015. In 2011 and 2013, 28 grantees completed the survey; all 29 grantees completed the survey in 2012 and 2015. Data source includes the CRCCP grantee survey data, 2011–2015.

**Table:** 

Intervention	No. of Grantees			
	2011	2012	2013	2015
Small media	27	28	28	25
Client reminders	21	22	23	26
Reducing structural barriers	14	17	23	23
Provider reminders	9	11	19	19
Provider assessment and feedback	14	13	15	17

### Perceived ease of EBI implementation

In general, the average ease of implementation ratings declined over time, indicating that grantees rated the same EBIs as more difficult to implement later in the program period (Table 2). This was not a perfect linear trend, yet every EBI had a lower average rating for program year 6 than for program year 2. The greatest decline was for provider assessment and feedback (1.18 points on a 5-point scale) and the smallest decline was for small media (0.23 points). Only grantees implementing a given EBI rated its ease in a given year. To see if the decline in average ease was due to those grantees newly implementing the EBIs each year, we restricted analysis to only those grantees who had implemented a given EBI the year before (ie, the “maintainers”; data not shown). In general, the average ratings of grantees implementing a given EBI for the first time and maintainers-only grantees were about the same (differences between the 2 groups were ≤.20 on a 5-point scale). The only exception was provider assessment and feedback; maintainers in program year 3 rated this EBI as more difficult (2.50) than new implementers (3.10).

### Grantees’ open-text responses

Grantees had the option to provide open-text responses describing EBI successes, challenges, and reasons why they no longer implement specific EBIs. The survey did not require responses to these items; therefore, a minority of grantees provided responses for each EBI in a given year (range, 2–12 grantees per EBI writing comments for EBI successes, challenges, or reasons for discontinuing each year). Across EBIs, there were 25 to 30 open-text responses each program year.

 Across EBIs, many grantees mentioned successful partnerships as key facilitators to implementation. In many cases, grantees’ partners led implementation activities; in others, they provided connections, materials, or staff time. Staff capacity, as well as having well-trained staff, were also listed as important facilitators. Several grantees also discussed electronic health records as a facilitator for EBIs that involved sending information to clients and for the provider-oriented EBIs.

Several of the facilitators were also described as barriers. Grantees discussed problems with specific partnerships, lack of staff time or capacity, and challenges working with their partners’ electronic health records as significant barriers to implementing EBIs. Other frequently mentioned barriers included getting approvals or arranging contracts with partner agencies and concerns about funding and sustainability. Grantees implementing provider-oriented interventions discussed competing provider/clinic priorities as a barrier.

Few grantees provided reasons for discontinuing specific EBIs. Of those who did, a common reason given (especially in program year 6) was the end of funding to sustain the EBI. A few grantees also noted that the EBI was part of a specific demonstration project within their CRCCP and that implementation ended when the demonstration project ended. The other most commonly given reasons for stopping specific EBIs included limited staff time or staff turnover and the desire to implement other EBIs (and not being able to implement all EBIs at one time). A couple of grantees also noted a shift in their partners’ focus or priorities that led to the partner no longer being interested in the EBI.

## Implications for Public Health

The first 6 years of the CRCCP provided a unique opportunity to study a consistent group of 29 organizations and how they adopted, implemented, and maintained or discontinued EBIs to promote CRC screening. We found that most grantees adopted and implemented small media and client reminders early in the study period; most grantees also maintained these 2 EBIs through 2015. These client-oriented EBIs are often considered simpler to implement than the provider-oriented EBIs because they do not necessarily require partnerships with health systems or modifications to electronic health records.

Adoption and implementation of the provider-oriented EBIs (provider reminders, provider assessment and feedback) and reduction of structural barriers was more gradual, with a few new grantees adopting these EBIs each year. There was also more turbulence in terms of implementation for these EBIs, with several grantees discontinuing them before 2015. Notable reasons for discontinuing EBIs were lack of resources, partners’ priorities, and ending of funding. These less-frequently implemented and sustained EBIs may have more potential to affect screening rates (15,16,21). However, provider-oriented EBIs are more complex, which can reduce implementation (22). In the second CRCCP cycle, CDC is requiring grantees to implement 2 or more of the following EBIs: client reminders, reducing structural barriers, provider reminders, and provider assessment and feedback in health system clinics (23). Our finding that 3 of these 4 EBIs were challenging to implement by the first cycle of CRCCP grantees highlights the importance of evaluating and better understanding their implementation efforts and challenges so as to inform development or dissemination of resources to make them easier to sustain. Grantees may still use small media, but only as an additional supporting strategy. This guidance is more specific and directs grantees to higher-impact EBIs from the beginning of the new funding cycle (24).

Our findings raise questions about the sustainability of provider-oriented EBIs as implemented by the grantees. Wiltsey Stirman and colleagues identified 4 influences on sustainability of new programs: context, characteristics of the new program (including complexity), processes, and capacity (9). We found that grantees’ reasons for discontinuing an EBI most often related to capacity issues. Context also may be an important factor. For instance, electronic health records systems were identified as a barrier to EBI implementation. This suggests that sustainability in clinic settings may be challenged when electronic health records systems cannot support integration of client and provider reminder systems as well as provider assessment and feedback reports. In the future, CRCCP and other similar programs may want to include more measures of sustainability and factors that influence sustainability (such as context) in evaluation instruments. These measures could potentially help grantees with their planning to sustain EBIs when funding ends and could help researchers better understand their implementation and de-implementation choices.

One of the counterintuitive findings of this study is that grantees did not find implementation easier with time. The trend appears to be that EBIs were perceived to be more difficult to implement over time, particularly for reducing structural barriers and provider assessment and feedback. Implementing the provider-oriented EBIs generally required strategic partnerships, and building and sustaining these partnerships is complex and takes ongoing effort (25). Challenges related to partnerships, electronic health records, and other issues may take a year or more to emerge. Another potential issue is that health systems serving high-need patients may find it difficult to maintain a focus on CRC screening, given competing priorities and limited resources.

Future research can explore determinants of EBI maintenance or abandonment and test strategies to assist organizations to maintain EBIs, including ones that address program factors, organizational context, processes, and capacity. CDC already provides training and technical assistance along with an incentive through CRCCP funding to implement EBIs. Additional strategies may be needed to help sustain more complex EBIs that require collaborating with health care systems and integrating with their health information technology. Future research should also explore implementation ease or difficulty in the context of the NBCCEDP, as grantees in this program are also encouraged to work with health system partners and use EBIs. There is significant overlap in NBCCEDP and CRCCP grantees, which creates the opportunity to discover potential synergies in implementing EBIs for multiple cancers across these programs or applying lessons learned from one program to another.

This study has several limitations. The small sample size of 29 grantees limited our ability to conduct inferential tests. This survey assessed only the grantees’ perspectives, whereas implementing the EBIs usually involved working with partner organizations who were not included in the survey. Some grantees experienced turnover in leadership such that the survey respondent changed over the course of the years studied; in these cases, it is difficult to know whether observed changes in the data reflect actual changes or changes in the perspective of the respondent. However, the pattern of rating implementation as stable or more difficult over time was consistent across years and across EBIs. Finally, not all grantees provided open-text data about barriers and facilitators to EBI implementation or their reasons for discontinuing EBIs. Given this, we cannot assess the overall impact of the barriers and facilitators on implementing and maintaining EBIs.

This study also has several strengths. We were able to study a group of 29 grantees that had stable funding over a 6-year period to implement EBIs to promote CRC screening, and we achieved very high response rates for the surveys across all years. The findings reveal not only which EBIs were adopted, but which were maintained over several years and thus had the most potential for sustained impact on screening rates. The findings have implications for the second CRCCP cycle (DP15–1502, 2015–2020) and for the implementation of EBIs in comparable clinical settings.
